# The Principles and Practice of Midwifery

**Published:** 1848-02

**Authors:** 


ARTICLE IX.

*The Principles and Practice of Midwifery.* By David H. Tucker, M. D., Professor of the Principles and Practice of Medicine, and formerly of Obstetrics, &c., in the Franklin Medical College, Philadelphia. (with numerous illustrations.) Philadelphia, Lindsay & Blakiston. 1848. pp. 485, 8vo. (From the Publishers.)

This work is the first of a series entitled "The Medical Practitioner's and Medical Student's Library," which the enterprising Publishers are preparing for publication. The several numbers of the series are to be published in "a cheap form, and at one half the usual price of Medical Books," thereby affording to the members of the Profession, possessing limited means, an opportunity of procuring valuable books at a small cost.

The work in question, is "a concise and practical treatise upon Obstetrics," compiled from the latest American,

English and French works, designed more particularly for the use of Medical Students, but is well deserving of a place in the Library of the Practitioner, as a book for reference and study.

The Publishers give notice that this will soon be followed by others of the series, treating upon the different Elementary and Practical Branches of Midwifery—each volume complete in itself.

From our examination of the work, we would recommend it, together with the Library, to members of the Profession in the West, as a valuable addition to our Medical Literature.

